# Iron Deposition following Chronic Myocardial Infarction as a Substrate for Cardiac Electrical Anomalies: Initial Findings in a Canine Model

**DOI:** 10.1371/journal.pone.0073193

**Published:** 2013-09-16

**Authors:** Ivan Cokic, Avinash Kali, Xunzhang Wang, Hsin-Jung Yang, Richard L. Q. Tang, Anees Thajudeen, Michael Shehata, Allen M. Amorn, Enzhao Liu, Brian Stewart, Nathan Bennett, Doron Harlev, Sotirios A. Tsaftaris, Warren M. Jackman, Sumeet S. Chugh, Rohan Dharmakumar

**Affiliations:** 1 Biomedical Imaging Research Institute, Cedars-Sinai Medical Center, Los Angeles, California, United States of America; 2 Department of Biomedical Engineering, Northwestern University, Evanston, Illinois, United States of America; 3 Department of Biomedical Engineering, University of California Los Angeles, Los Angeles, California, United States of America; 4 Heart Institute, Cedars-Sinai Medical Center, Los Angeles California, United States of America; 5 Rhythmia Medical, Burlington, Massachusetts, United States of America; 6 Institutions Markets Technologies, Institute for Advanced Studies Lucca, Piazza S. Ponziano, Lucca, Italy; 7 Department of Electrical Engineering and Computer Science, Northwestern University, Evanston, Illinois, United States of America; 8 Department of Radiology, Northwestern University, Chicago, Illinois, United States of America; 9 Heart Rhythm Institute, University of Oklahoma Health Sciences Center, Oklahoma City, Oklahoma, United States of America; 10 Department of Medicine, University of California Los Angeles, Los Angeles, California, United States of America; University of Illinois at Chicago, United States of America

## Abstract

**Purpose:**

Iron deposition has been shown to occur following myocardial infarction (MI). We investigated whether such focal iron deposition within chronic MI lead to electrical anomalies.

**Methods:**

Two groups of dogs (ex-vivo (n = 12) and in-vivo (n = 10)) were studied at 16 weeks post MI. Hearts of animals from ex-vivo group were explanted and sectioned into infarcted and non-infarcted segments. Impedance spectroscopy was used to derive electrical permittivity (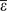) and conductivity (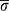). Mass spectrometry was used to classify and characterize tissue sections with (IRON+) and without (IRON-) iron. Animals from in-vivo group underwent cardiac magnetic resonance imaging (CMR) for estimation of scar volume (late-gadolinium enhancement, LGE) and iron deposition (T2*) relative to left-ventricular volume. 24-hour electrocardiogram recordings were obtained and used to examine Heart Rate (HR), QT interval (QT), QT corrected for HR (QTc) and QTc dispersion (QTcd). In a fraction of these animals (n = 5), ultra-high resolution electroanatomical mapping (EAM) was performed, co-registered with LGE and T2* CMR and were used to characterize the spatial locations of isolated late potentials (ILPs).

**Results:**

Compared to IRON- sections, IRON+ sections had higher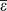, but no difference in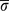. A linear relationship was found between iron content and 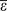 (p<0.001), but not 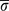 (p = 0.34). Among two groups of animals (Iron (<1.5%) and Iron (>1.5%)) with similar scar volumes (7.28%±1.02% (Iron (<1.5%)) vs 8.35%±2.98% (Iron (>1.5%)), p = 0.51) but markedly different iron volumes (1.12%±0.64% (Iron (<1.5%)) vs 2.47%±0.64% (Iron (>1.5%)), p = 0.02), QT and QTc were elevated and QTcd was decreased in the group with the higher iron volume during the day, night and 24-hour period (p<0.05). EAMs co-registered with CMR images showed a greater tendency for ILPs to emerge from scar regions with iron versus without iron.

**Conclusion:**

The electrical behavior of infarcted hearts with iron appears to be different from those without iron. Iron within infarcted zones may evolve as an arrhythmogenic substrate in the post MI period.

## Introduction

The electrical behavior of chronically infarcted myocardium is not well understood. Conventionally, infarcted myocardium is identified on electroanatmoical maps (EAMs) on the basis of significantly reduced bipolar voltage (below 0.5 mv) [1], [2]. While a significant portion of the scarred myocardium is thought to be electrically inert, the presence of non-zero voltage points within the infarct zone has been associated with surviving myocytes [3], [4]. However, several other studies have shown direct evidence for significant passive electrical activity within the dense scar that is free of viable myocytes [5], [6]. Nevertheless, the substrate mediating the electrical activity within the infarcted tissue devoid of surviving myocyte bundles remains to be explored.

Observational studies in patients with pathological iron (hemosiderin) overloading in the heart, from non-ischemic origins (hemochromatosis [7], [8], thalassemia [9], [10], siderosis [11], and sickle-cell anemia [12]), have long documented evidence of significant incidence of ventricular arrhythmias [13]–[16]. Imaging studies in the same patient population have also suggested that the incidence of arrhythmias to be directly related to the extent of myocardial iron deposition [16]. Moreover, carefully controlled animal studies have shown that cardiac iron overloading leads to progressively worsening electrical conductivity with increasing iron (hemosiderin) deposition, even in the absence of myocardial contractility changes [14]. Notably, these studies showed that approximately 1 in 3 animals with chronic iron overloading succumbed to sudden cardiac death (SCD) attributable to cardiac arrhythmias. More recently, magnetic resonance imaging based histological examination of human hearts of SCD victims have shown significant loss of T2-weighted signals within the chronic infarcted territories, consistent with hemosiderin accumulation [17], [18].

The relationship between myocardial iron deposition and electrical changes may be explored on the basis of existing biophysical findings. It has been shown that the introduction of highly conductive particulates (such as magnetite with conductivity of 2.5×10^4^ S/m at the physiologic temperature) [19] into an otherwise poor dielectric medium (such as the myocardium with conductivity <1 S/m) [20] acts to enhance the bulk electrical permittivity of the medium [21], [22]. Hence, the pathological elevations of iron within localized regions of the heart may be a substrate that alters the electrical milieu of myocardial regions containing iron.

Recent studies in humans and animals have demonstrated that chronic iron (hemosiderin) overloading within the scar tissue may occur following myocardial infarctions due to pooling of blood within the infarcted territories [23]. Based on biophysical principles alone, it is expected that the electrical permittivity of post-infarction scar with iron deposits can be significantly greater than those scars without iron deposits. If such changes in the electrical features of the infarcted myocardium manifest in the same manner as in the case of iron overloading from the non-ischemic pathologies, one is expected to observe global and local electrical changes that are different between infarcted hearts with and without iron depositions.

Through controlled experiments in a canine model, this study investigates whether infarcts with chronic iron deposition, identified on the basis of mass spectrometry, preferentially alters the electrical features of myocardial tissue in ex-vivo preparations. In addition, it also examines whether there are differences in global and local electrical characteristics of the infarcted hearts with different levels of iron deposition, determined on the basis of cardiac magnetic resonance imaging (CMR). In particular, this study explores whether the established parameters derived from surface electrocardiograms (ECG) and EAMs are altered in a manner that is dependent on the extent of iron deposition.

## Methods

### Ethics Statement

Mongrel dogs were studied according to the protocols approved by the Animal Care and Use Committee of Northwestern University (IACUC number: 2007–1286) and Cedars-Sinai Medical Center (IACUC number: 0003674). The Animal Care and Use Committee of Northwestern University and Cedars-Sinai Medical Center approved the studies described below.

### Animal Preparation and Overview of Methods

Myocardial infarction (MI) was created by permanently ligating the left anterior descending (LAD) artery distal to the first diagonal in 22 dogs (20–25 kg), and allowed to recover for 16 weeks. In the first group (Group *ex-vivo*, n = 12), 10 animals survived into the chronic phase of the infarction and 2 animals died during the acute phase of MI. Animals from Group *ex-vivo* were sacrificed and their hearts were harvested, and sectioned into 1 cm thick slices. Infarcted and non-infarcted (Remote) segments on each slice were delineated on the basis of *ex vivo* triphenyl tetrazolium chloride (TTC) staining [24], and 0.5–0.8 cm^3^ samples of tissue were isolated from the respective segments. Representative samples were selected for histological staining (Perl's stain). Samples from infarcted segments were further classified as those with and without iron deposition (IRON+ and IRON− respectively) based on mass spectrometric analysis (details below). Prior to mass spectrometric analysis, bulk electrical impedance spectroscopy measurements were performed on tissue samples from all the three groups (IRON+, IRON− and Remote).

The animals from Group *in-vivo* were fitted with a Holter monitor and ECG measurements were recorded over a 24-hour period (details below). Subsequently the animals from this group underwent CMR studies (within a week of the ECG recordings) to determine the extent of iron deposition within the myocardial infarct territories (details below). Following CMR exams, 5 animals from this group underwent ultra-high resolution endocardial left ventricular electroanatomical mapping (EAM, details below).

### Ex vivo Bulk Electrical Impedance Measurements

**Figure 1:**
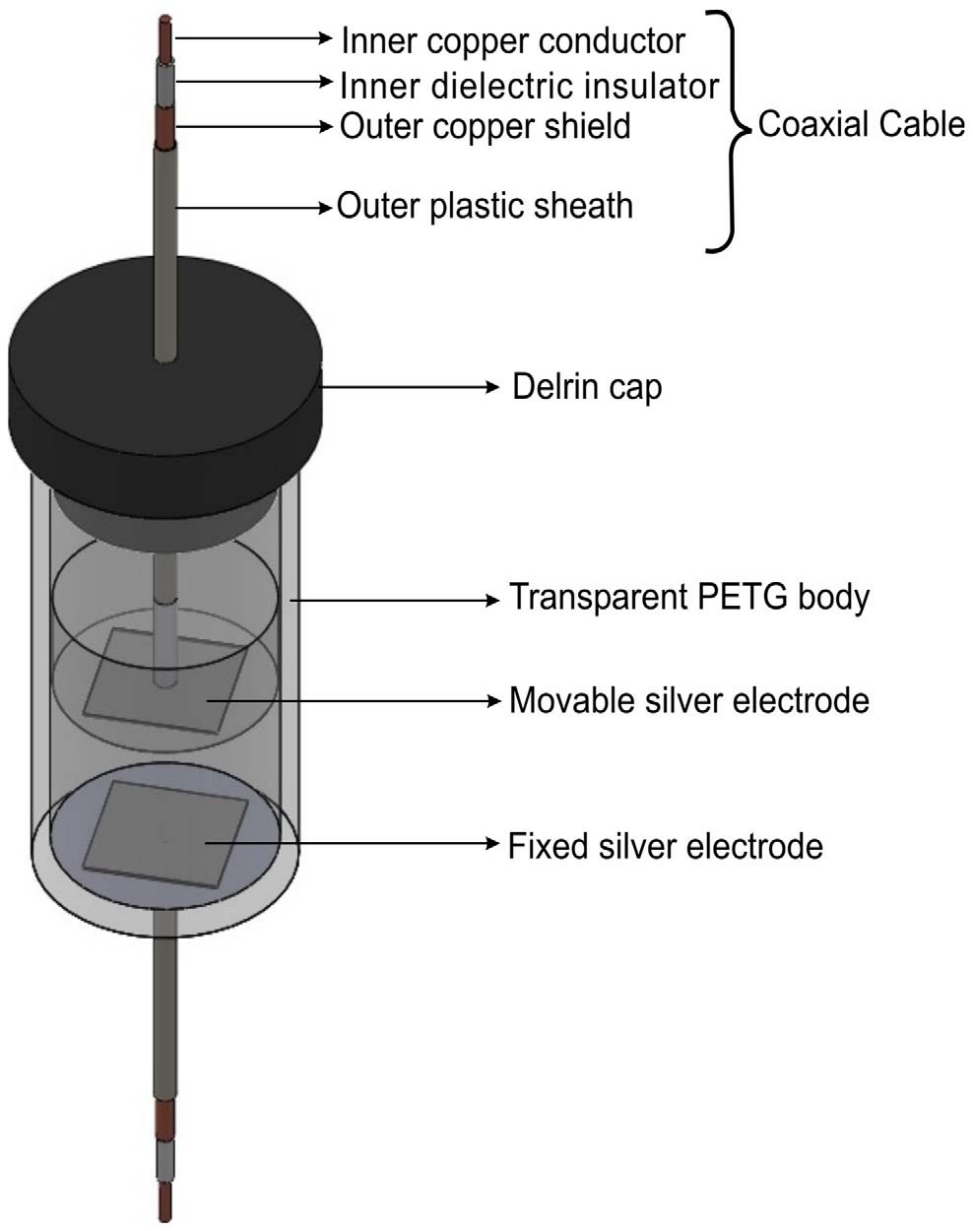
Schematic three-dimensional drawing of a custom-made capacitor cell used for measuring bulk electrical impedance of ex-vivo tissue. The capacitor cell consisted of a transparent tubular glycol-modified polyethylene teraphthalate (PETG) body that is closed at one end and fitted with a removable Delrin cap at the other end. Two square silver electrodes, each of 1.5 cm^2^ surface area, were enclosed in the tubular body. One electrode was affixed to the closed end, while the other electrode was affixed to a PETG disk that can move through the tubular body. The electrodes were soldered to the inner conductors of copper coaxial cables, which in turn were connected to the impedance analyzer. The outer conductors were connected to electrical ground.

The bulk electrical impedance of each tissue sample was measured using the two-terminal electrode technique as previously described by Schwan [25]. A capacitor cell consisting of two parallel square electrodes (each of 1.5 cm^2^ surface area) with variable distance between them [26] was custom built to measure bulk electrical impedance of each sample using alternating-current (AC) impedance spectroscopy (Fig. 1).

Each sample was incubated at 37°C in phosphate buffer solution for 15 minutes prior to use. The sample was then sandwiched between the two electrodes of the capacitor cell and 10 µA of alternating current was passed parallel to the myocardial fibers. The voltage that developed across the sample was measured using Solartron 1260 impedance/gain-phase analyzer (Solartron Instruments, Hampshire, UK) and acquired using ZPlot data acquisition software (version 3.3, Scribner Associates Inc., NC, USA). The complex AC-impedance (*Z* in ohms) of the sample was estimated as a quotient of the induced voltage and the current supplied to the sample. The impedance values were measured at frequencies ranging from 100 Hz to 10 MHz with 10 measurements in each frequency decade. Stray effects in the measurements were corrected using methods described by Schwan [25]. To minimize the effects of α-dispersion at lower frequencies (below 0.1 MHz) and β-dispersion (above 1 MHz) [27] from undesirable myocardial sample preparation errors (such as, partial volume effects from samples containing both infarcted tissue and surrounding viable myocardium), all analyses were limited to impedance data acquired at 1 MHz. Based on the impedance measurements, electrical permittivity and conductivity were estimated as previously described [28]. Refer to File S1.

For a given heart from an infarcted dog, mean conductivity (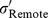) and permittivity (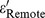) of its remote myocardium were calculated by weight-averaging the conductivities and permittivities of all of its constituent remote samples as follows:
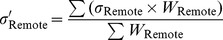


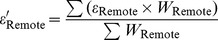
where, 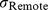
and 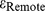
are the individual conductivity and permittivity of each constituent remote sample of a heart and 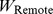 is its corresponding sample weight. Normalized conductivity (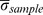) and permittivity (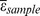) of each IRON+, IRON− and Remote sample from the heart were then derived as follows:



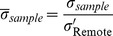


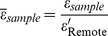



Also, per-slice normalized conductivity (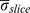) and permittivity (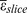) were calculated for the IRON+, IRON−, and Remote groups by weight averaging 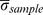 and 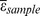 respectively from their constituent samples.

### Inductively Coupled Plasma – Mass Spectrometry (ICP-MS)

Iron deposition within each myocardial sample from Group *ex-vivo* ([Fe]*_sample_* in mg per g of tissue) was measured using a quadrupole-based X Series 2 ICP-MS (Thermo-Fisher Scientific, USA). Infarcted sections with [Fe]*_sample_* <0.05 mg/g [23] of tissue were labeled as IRON− and those with [Fe]*_sample_* ≥0.05 mg/g were labeled as IRON+. In all, 293 sections from a total of 11 dogs were analyzed, where 228 sections were infarcted and 67 sections were non-infarcted.

### Holter ECG Recordings and Surface ECG Analysis

24-hour Holter ECG recordings were made 16 weeks post MI in animals from Group *in-vivo* (n = 10) using a three-channel recorder (DigiTrak XT Holter Recorder, Philips Zymed Holter System, Philips Healthcare, MA, USA). Protective bandages and custom-made jackets were used to secure the Holter over the animals. The derived 12-lead ECG was recorded continuously using five adhesive leads placed (a) over the lower sternum, level with the fifth intercostal space; (b) at the level of the fifth intercostal space, on the left midaxillary line; (c) on the upper part of the sternum; (d) at the fifth intercostal space, on the right midaxillary line; and (e) on the manubrium of the sternum (reference/ground electrode).

All ECG recordings underwent automated retrospective analysis using the dedicated Holter software (DigiTrak XT Holter Recorder, Philips Zymed Holter System Philips Healthcare, MA, USA). The analysis of heart rate (HR) and repolarization parameters (QT, QTc) was performed after adjusting all recordings to the 24-hour clock. Only recordings with duration of 24 hours were considered for analysis. An operator blinded to the MR details of the infarct characteristics analyzed the Holter recordings. The morphology of the beats was monitored and only normal beats (sinus rhythm) were selected for further analysis. ECG segments with excessive noise or artifact were eliminated from the analysis. The hourly mean values of each measurement were automatically calculated. QT intervals corrected for heart rate (QTc) were automatically calculated using Bazett's formula [29]. QTc dispersion (QTcd) was computed as the difference between the maximum and the minimum QTc interval across the 12 derived leads.

### CMR Exam and Image Analysis

Prior to imaging, canines were fasted for 18 hours, sedated, anesthetized, intubated, transferred to the scanner table and were connected to a ventilator for mechanical ventilation. Animals were maintained on gas anesthesia (2–2.5% isoflurane and 100% oxygen). CMR studies were performed on a clinical 3T system (MAGNETOM Verio, Siemens Medical Solutions, Erlangen, Germany) within one week of ECG recordings being taken. Multi-gradient echo T2*-weighted (TR  = 12 ms, 6 TEs  = 2.0 ms –9.5 ms with ΔTE  = 1.5 ms, flip angle  = 10° and BW  = 930 Hz/pixel) and late-gadolinium enhancement (LGE) images (inversion-recovery prepared FLASH, TR / TE  = 3.0/1.5 ms, flip angle  = 25° and BW  = 586 Hz/pixel) of contiguous short-axis sections covering the entire left ventricle (LV) and the three long-axis views were acquired at mid-diastole. Commonly used imaging parameters were: in-plane resolution  = 1.3×1.3 mm^2^, slice thickness  = 6 mm and number of averages  = 1.

Image analyses were performed using cmr^42^ (v4.0, Circle Cardiovascular Imaging Inc., Canada). Remote myocardium was defined as the region showing no hyperintensity on LGE images. A reference region-of-interest (ROI) was drawn in the remote myocardium on both LGE and T2*-weighted images. Infarcted territory was defined as the hyperintense region on LGE images with mean signal intensity (SI) of at least 5 standard deviations (SD) above that of the reference ROI [30]. An infarction with chronic iron deposition (IRON+) was defined as infarcted territory containing hypointense signal on the T2*-weighted image acquired at TE of 6.5 ms with mean SI of at least 2SDs below that of the reference ROI [31], [32]. An infarction without iron deposition (IRON−) was defined as the region positive for infarction on LGE images, but negative for hypointense signal on the corresponding T2*-weighted images. The percentage of the infarcted myocardium (*Scar Volume*) and myocardium with chronic iron deposition (*Iron Volume*) were computed by summing up the respective slice measures and normalizing by the total left-ventricular volume.

### Electroanatomical Mapping, Analysis and Registration with CMR Images

Electrophysiological studies were performed within 3–7 days of the CMR studies in 5 animals under general anesthesia and mechanical ventilation (protocol same as during CMR). The left femoral artery was cannulated, and a 64-electrode basket catheter (nominal diameter 18 mm) with 8 splines, each containing 8 tiny electrodes (0.4 mm^2^) spaced at 2.5 mm, center-to-center (Rhythmia Mapping System, Boston Scientific, MA, USA) was introduced into the left ventricle using the retro-transaortic approach. The catheter was used to generate ultra-high resolution endocardial contact electrograms (EGMs; within 2–3 mm spatial resolution). The surface geometry was mapped using the location of the outermost electrodes. Only EGMs recorded within 2 mm of the surface geometry were utilized. Scar areas were defined as bipolar voltage less than 1 mV [33].

At least three clinical electrophysiology experts analyzed the EAMs from each animal offline. Given the large number of data points within and surrounding the scarred myocardium, only points separated by 1.5–2 mm of one another were manually validated. The validation process included ensuring catheter contact and identifying and marking each point of interest for the presence or absence of isolated late potentials (ILPs). An ILP was defined as a voltage spike following an isoelectric interval observed after the end of the QRS complex [34]–[36].

The LGE and T2* CMR data were registered with the EAMs using a custom-developed software. The registration process involved (a) manually segmenting the blood pool of both LGE and T2* images; (b) constructing a surface that encloses the segmented blood pool; and (c) manually registering both the LGE and T2* blood pool surface to the EAM surface using anatomical landmarks (apex, papillary muscle grooves and aortic root). The gray scale CMR data was resampled at the vertices of the CMR blood pool mesh and displayed as a colored 3D surface with colors corresponding to signal intensity.

After registration with the EAMs, the T2* blood pool mesh was used to manually count the number of ILPs occurring in and around the infarcted regions with and without iron. This information was used to derive values for the overall incidence of ILPs, which were computed as a percentage of ILPs from regions with and without iron relative to the total number of ILPs in the infarct territory. To examine the relationship between the number of ILPs and substrate type and extent, values normalized by the volume fraction of substrate type in each heart were computed and averaged across all animals. In particular, the following calculations were performed: (a) ILP counts from regions with iron normalized by *Iron Volume*; (b) ILP counts from regions without iron normalized by the percentage scar volume without iron (i.e. *Scar Volume* – *Iron Volume*); and (c) the total ILP count normalized by the percentage of total scar volume (with and without iron) relative to the total volume of myocardium.

From the EAMs registered with T2* images, number of ILPs occurring in and around the infarcted regions with and without iron were manually counted. This information was used to derive the overall incidence of ILPs, which was computed as a percentage of ILPs from regions with and without iron relative to the total number of ILPs. To examine the relationship between number of ILPs and substrate type and extent, values normalized by the volume fraction of substrate type in each heart were computed and averaged across all animals. In particular, the following calculations were performed: (a) ILP counts from regions with iron normalized by *Iron Volume*; (b) ILP counts from regions without iron normalized by percentage scar without iron (i.e. *Scar Volume* – *Iron Volume*); and (c) the total ILP count normalized by *Scar Volume* (i.e. myocardial volume normalized scars with and without iron).

### Statistical Analysis

All statistical analyses were performed using SPSS (IBM SPPS Statistics v21.0, New York, NY, USA). A p value <0.05 was considered statistically significant. Bonferroni correction was used to adjust the significance level for multiple comparisons. Data normality was assessed using the Shapiro-Wilk test and quantile-quantile plots. Student's t-test or mixed-model ANOVA with Tukey's post-hoc analysis was used to compare data with normal distributions. For the mixed model, animals were entered as random effects, while repeated measurements from the same animal or heart were entered as fixed effects. Repeated measurements from a single animal or heart were nested. For comparing non-normal data, non-parametric Friedman's test was used. Mann-Whitney U test was used for pairwise comparisons among non-normal data.

For the canines from Group *ex vivo*, 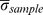 and 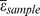 were compared among IRON+, IRON−, and Remote tissue sections. Mixed-model linear regressions was used to evaluate the relationships of 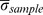 and 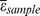 with [Fe]*_sample_*. A nonlinear regression analysis was used to examine the relationship between *Iron Volume* and *Scar Volume*.

In order to examine the effect of *Iron Volume* on the ECG parameters (HR, QT, QTc and QTcd) with minimal/no contribution from *Scar Volume*, animals were divided into two groups, those with *Iron Volume* <1.5% (denoted as Iron (<1.5%)) and those with *Iron Volume* >1.5% (denoted as Iron (>1.5%)). Only those animals that showed a direct relationship between *Iron Volume* and *Scar Volume* were included in the analysis (details below). Mean hourly, day (10∶00 hrs to 21∶00 hrs), night (21∶00 hrs to 09∶00 hrs) and 24-hour measurements of HR, QT, QTc, and QTcd were computed for Iron (<1.5%) and Iron (>1.5%) groups and compared, as above.

Regression analysis was used to assess the quality of the registration between CMR and EAMs on the basis of concordance between scar territories identified on LGE CMR and low voltage (<1 mV) vertices on bipolar EAMs. The incidence of ILPs was compared between scar regions with iron and without iron. The number of ILPs per volume of substrate for scars with only iron and without iron was also compared.

## Results

### Effect of Iron Deposition on the Ex-vivo Electrical Capacitance of Myocardial Infarcts

**Figure 2:**
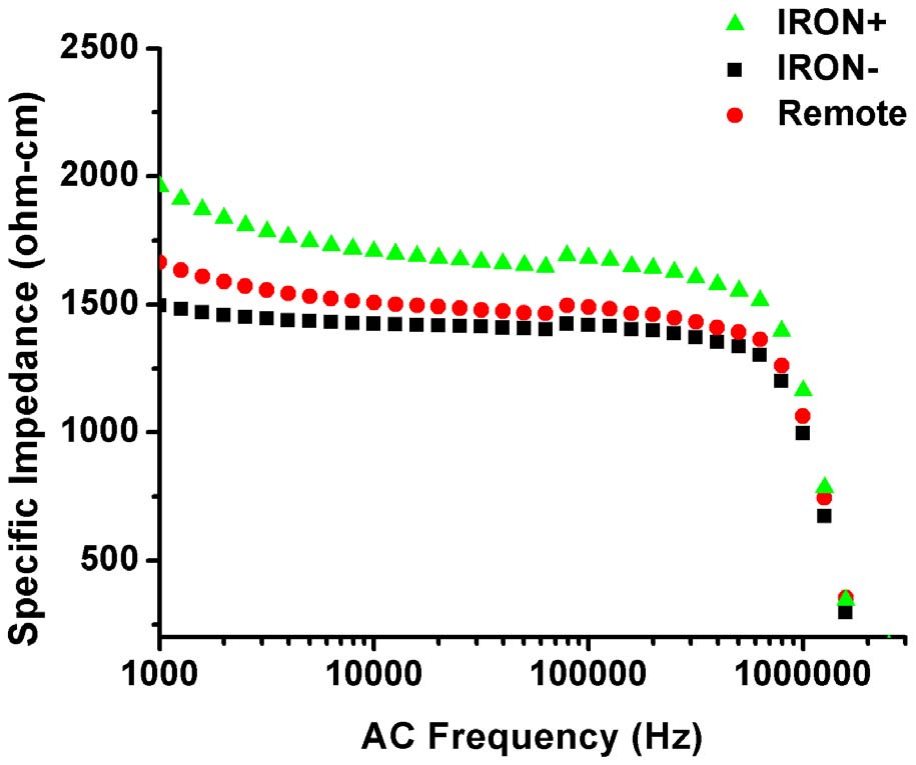
Representative specific impedance spectra from Remote, IRON-, and IRON+ myocardial samples. Note that for a given AC frequency, the specific impedance of IRON+ samples is higher than that of the Remote and IRON− samples.

**Figure 3:**
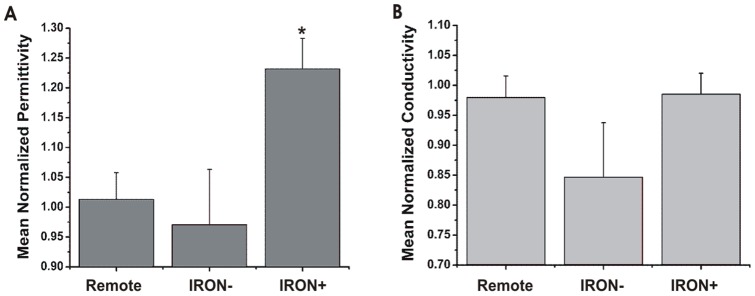
Electrical consequences of iron deposition in ex-vivo myocardium. (**A**) Mean 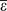 measured from Remote, IRON−, and IRON+ infarct sections showed significantly greater 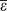 (*, p<0.001) in IRON+ compared to Remote and IRON− sections; (**B**) however, mean 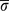 measured from Remote, IRON−, and IRON+ infarct sections did not show any statistical difference in 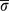 between the different sections.

Of 228 infarcted sections, mass spectrometry analysis identified 177 sections as IRON+ and the remaining 51 sections as IRON−. There was no significant difference in [Fe]*_sample_* between IRON− and Remote samples (p = 0.31). A set of sample impedance spectra from IRON+, IRON−, and Remote sections from an animal are shown in Fig. 2. Mean 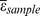 for IRON+ sections was significantly different from mean 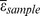 for IRON− and Remote tissues (p<0.001), while 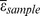
of IRON− and Remote sections were not statistically different from 1 (p = 0.69; Fig. 3A). Mean 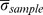 was not significantly different among the different tissue types (Remote vs IRON+: p = 0.46; Remote vs IRON−: p = 0.77; Fig. 3B). Averaged across all studies, a mean increase in 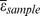 of approximately 25% in the infarcted territories with iron deposition was observed, while no change was observed in remote or infarcted territories without iron. Mixed-effects multi-linear regression analysis showed a statistically significant relation between 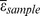 and [Fe]*_sample_* (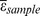  = 1.34 [Fe]*_sample_* +0.93; p<0.001; Table 1), but not between 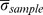 and [Fe]*_sample_* (p = 0.34; Table 2).

**Table 1:** Relationship between Normalized Permittivity and Iron Content.

Normalized Conductivity	Coefficient	Standard Error	Z	P>Z	Lower 95% CI	Upper 95% CI
[Fe] in mg/g of tissue	0.23	0.24	0.96	0.337	−0.24	0.71
Constant	0.86	0.09	9.97	<0.001	0.69	1.02

**Table 2:** Relationship between Normalized Permitivity and Iron Content.

Normalized Permittivity	Coefficient	Standard Error	Z	P>Z	Lower 95% CI	Upper 95% CI
[Fe] in mg/g of tissue	1.34	0.31	4.33	<0.001	0.73	1.95
Constant	0.94	0.10	9.30	<0.001	0.74	1.13

### In-vivo Studies

**Figure 4:**
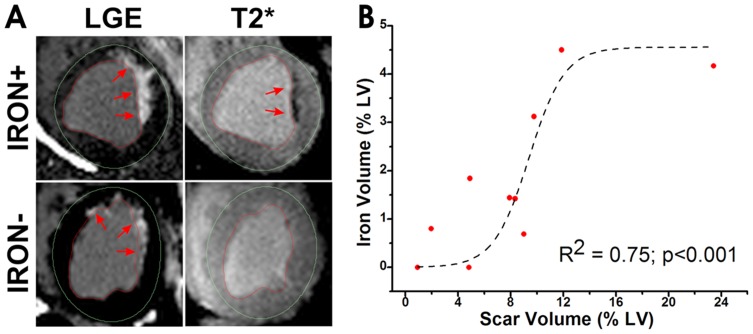
Relation between scar features and chronic iron deposition. (**A**) Representative short-axis LGE and T2*-weighted (TE  = 6.5 ms) images from two canines subjected to MI from Group in vivo – one with chronic iron deposition within the scar territory (Iron (>1.5%)) and another without chronic iron deposition (Iron (<1.5%)) are shown. Red arrows point to the site of myocardial scar on the LGE images in both the cases and to chronic iron deposition on the T2*-weighted image. (**B**) A significant sigmoidal relation was found between scar volume and iron volume (both computed as a percentage of total LV myocardium; R^2^ = 0.75, p <0.001).

**Figure 5:**
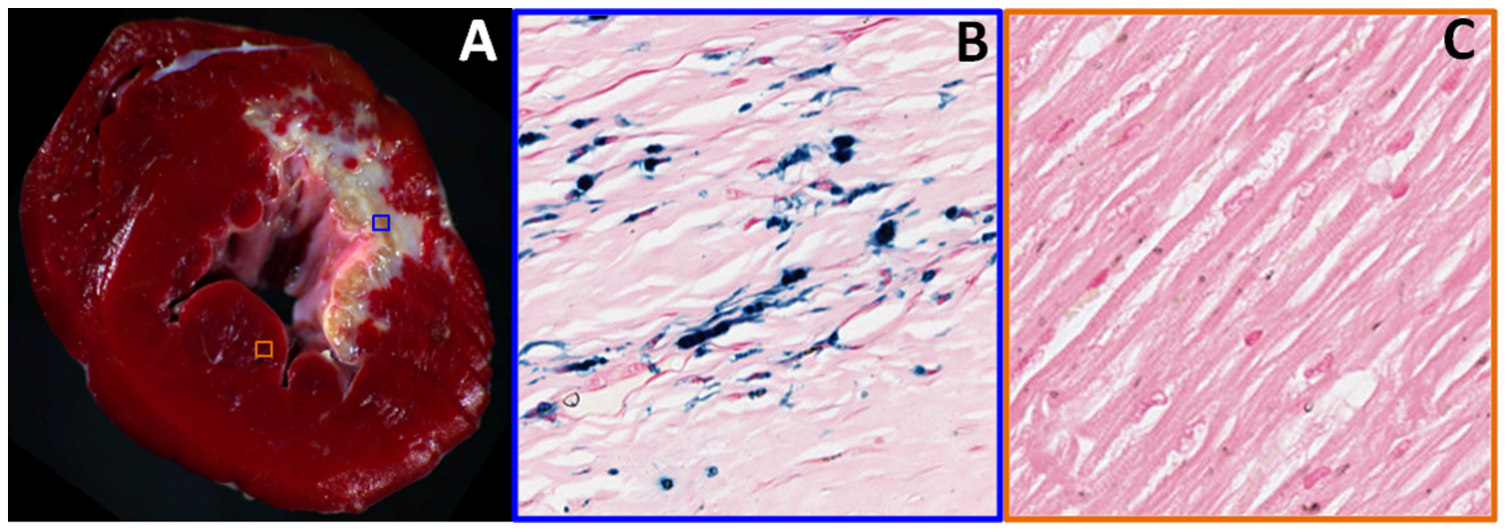
Histological Findings. The presence of infarction apparent on TTC staining (**A**) and iron (**B**, blue Perl's stains; black arrows) within chronic MI and its absence (**C**, Perl's stain) in remote sections are shown. Note that the iron deposits are typically found in the interstitial/extracellular space. The blue and red boxes within the TTC images correspond to the histology sections from infarcted and remote (non-infarcted) regions.

All 10 animals were positive for infarction and survived the CMR study. CMR studies showed iron depositions to be within the infarcted tissue. Specifically, the imaging studies showed that the iron deposition began at the subendocardium but did not extend beyond the mid wall. In addition, regression analysis between the *Scar Volume* and *Iron Volume* showed a sigmoidal relationship (R^2^ = 0.75, p<0.001, Fig. 4), indicating that *Iron Volume* is small when *Scar Volume* is small (<5%), rapidly increasing at intermediate levels of scar (5–12%) and reaching a plateau with any additional increases in *Scar Volume*. Representative results from gross TTC staining and histological staining for iron are shown in Fig. 5.

#### Surface ECG Recordings

**Figure 6:**
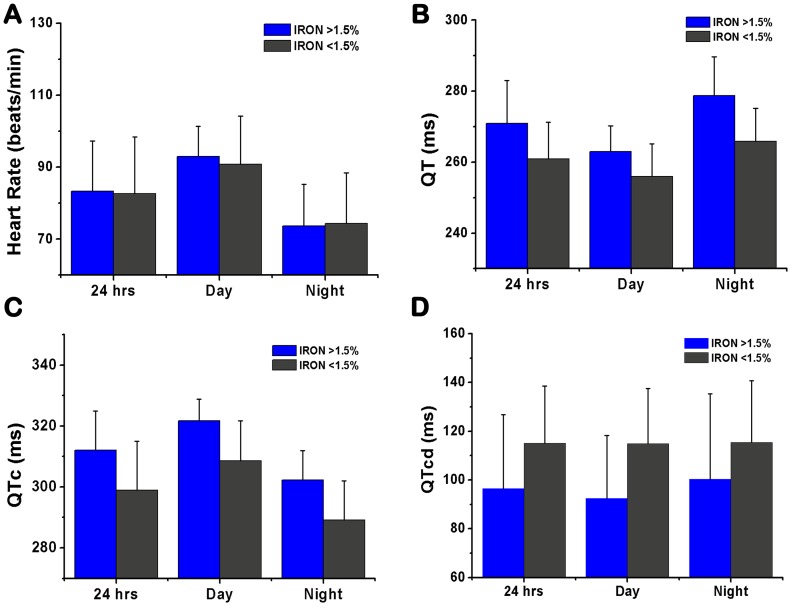
Mean values of important surface ECG parameters over day, night and a 24-hour period from Iron (>1.5%) and Iron (<1.5%) dogs. The mean values from dogs with and without iron over the period of interest for heart rate (**A**), QT (**B**), QTc (**C**) and QTcd (**D**) are shown.

**Figure 7:**
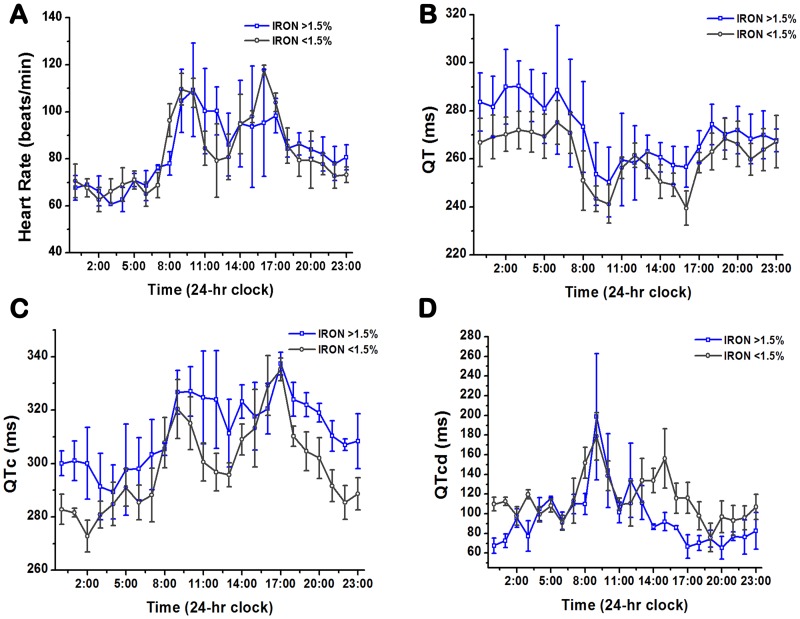
24-hour Holter ECG recordings from Iron (>1.5%) and Iron (<1.5%) dogs. The mean 24-hour traces showing changes in heart rate (**A**), QT (**B**), QT corrected for heart rate (**C**), and QTc dispersion (**D**) are shown for the two different groups of dogs with and without iron deposition.

To minimize the confounding effects of Scar Volume on ECG parameters [37], [38], in total 7 out of the 10 animals that underwent surface ECG recordings (n = 3 in Iron (<1.5%) and n = 4 in Iron (>1.5%)) were studied. The animals with the extreme values of Scar Volume (i.e. on the plateau regions of the sigmoidal response curve, Fig. 4) were not included as part of the analysis. The mean Iron Volume of the two groups were: 1.12%±0.64% (Iron (<1.5%)) vs 2.47%±0.64% (Iron (>1.5%)), p = 0.02; and the mean Scar Volume of the two groups were: 7.28%±1.02% (Iron (<1.5%)) vs 8.35%±2.98% (Iron (>1.5%)), p = 0.51. Mean HR, QT, QTc, and QTcd values obtained during day, night, and over 24 hours are shown in Fig. 6. Mean hourly tracings of HR, QT, QTc, and QTcd over the 24-hour period are shown in Fig. 7. QT, QTc and QTcd showed statistically significant differences between Iron (<1.5%) and Iron (>1.5%) groups. In particular, mean HR was not different between Iron (>1.5%) and Iron (<1.5%) groups during the day (77.9±17.0 beats/min vs 78.1±17.1 beats/min, p = 0.51), night (88.7±7.4 beats/min vs 86.9±13.4 beats/min, p = 0.73), or 24-hour period (83.3±14.0 beats/min vs 82.6±15.8 beats/min, p = 0.72). Mean QT was significantly different between Iron (>1.5%) and Iron (<1.5%) during the day (263.0±7.2 ms vs 255.9±9.17 ms, p<0.001), night (278.8±10.9 ms vs 265.9±9.3 ms, p<0.001), and 24-hour period (270.9±12.1 ms vs 260.9±10.4 ms, p<0.001). Similarly, mean QTc was significantly different between Iron (>1.5%) and Iron (<1.5%) during the day (321.8±7.1 ms vs 308.9±13.1 ms, p<0.001), night (302.3±9.5 ms vs 289.1±12.8 ms, p<0.001), and 24-hour period (312.0±12.9 ms vs 298.9±16.1 ms, p<0.001). In addition, mean QTcd was also significantly different between Iron (>1.5%) and Iron (<1.5%) during the day (92.4±25.8 ms vs 114.9±9.17 ms, p<0.01), night (100.4±35.0 ms vs 115.4±25.3 ms, p = 0.04), and 24-hour period (96.4±30.3 ms vs 115.1±23.5 ms, p<0.001).

#### Electroanatomical Mapping Measurements

**Figure 8:**
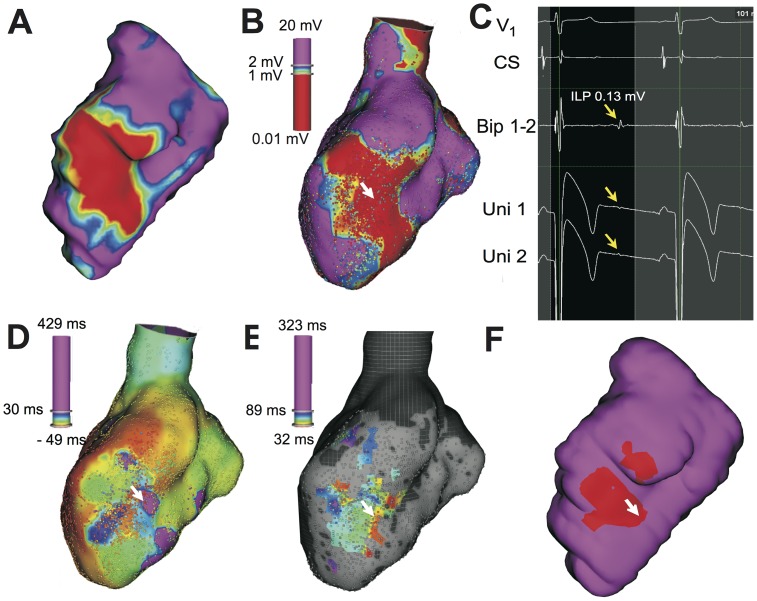
Representative co-registered CMR images and endocardial EAMs showing the association between ILPs and iron deposition following myocardial infarction. Co-registered late-gadolinium enhancement images projected onto the segmented blood pool surface (**A**) with infarcted territory (color coded in red), border zone (yellow and blue shades) and remote territories (purple)) with the corresponding bipolar map (**B**, color-coded to indicate low voltage areas) are shown. For reference, an ILP deep within the scar tissue (white arrow) is shown. The voltage traces from V1 and at the coronary sinus (CS), along with bipolar and unipolar mapping traces are also shown. Note the presence of an isolated low-voltage sharp late potential in the bipolar and unipolar traces following the local ventricular activation (yellow arrow) in **C**. The activation map (**D**), a map of the ILPs (**E**), and iron containing regions (in red, **F**) are also shown for reference. Note that iron-containing regions have a greater incidence of ILPs and slow activation regions.

**Figure 9:**
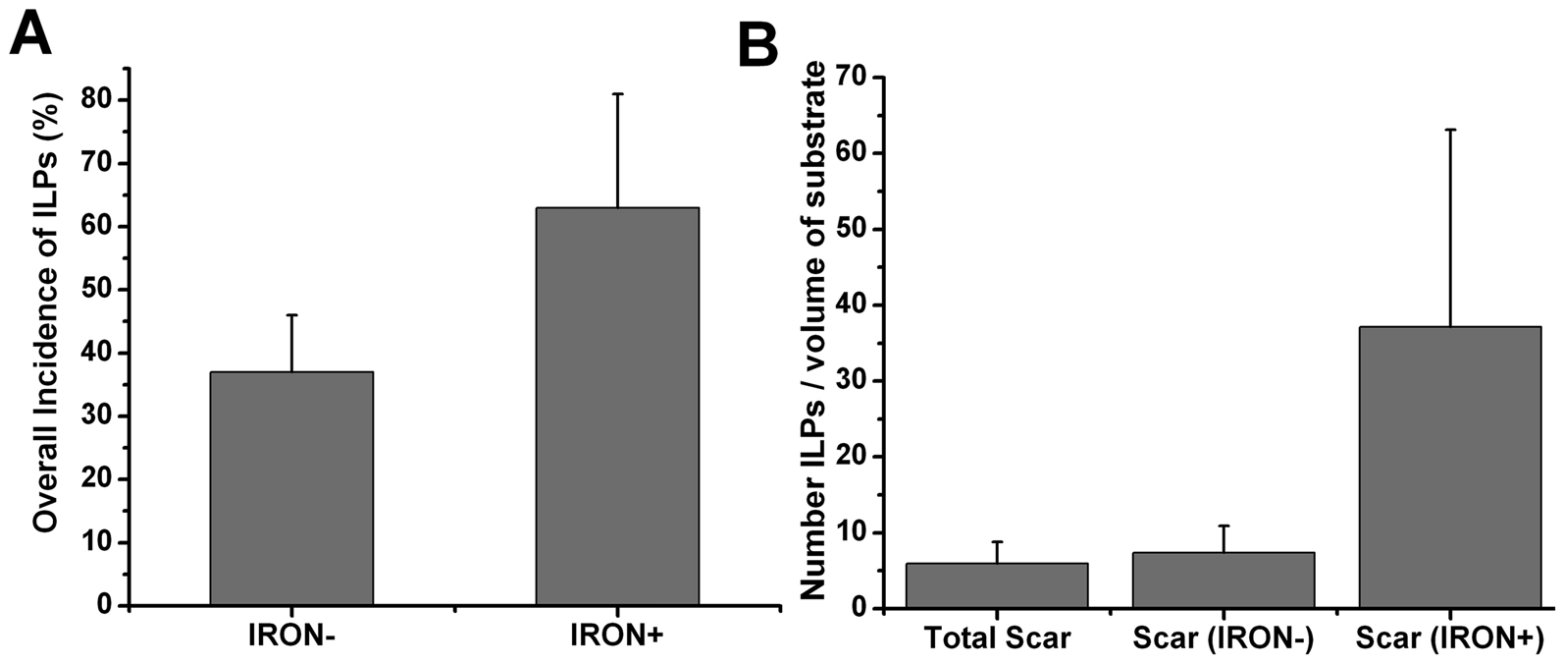
Dependence on the probability of observing ILPs based on substrate type and number of ILPS relative to substrate burden visualized on the basis of co-registered EAM and CMR images (LGE and T2*). (**A**) Shows the overall incidence of ILPs (fraction of the total) that were coincident with regions containing iron (IRON+) and regions without iron (IRON−). (**B**) Shows the mean number of ILPs per volume of substrate, with the substrate being the total scar (i.e., scar with and without iron), scarred regions with iron (IRON+) and scarred regions without iron (IRON−).

Of the 5 animals that underwent EAMs, one animal died during the mapping procedure from spontaneous and sustained ventricular tachycardia, despite cardioversion efforts. Regression analysis of the scar size and location performed between CMR and EAM were highly correlated (R^2^ = 0.77, p<0.05), indicating that the registration between the two imaging modalities was significant. Averaged across all animals, 7705±2212 electrograms (EGMs) were recorded and 1577±982 EGMs were manually validated on each map in and around the scar. A representative set of EAMs (bipolar voltage, bipolar activation and ILP maps) co-registered with CMR (LGE and T2*) is shown in Fig. 8. On average, a total of 158±79 ILPs were observed in each dog, and 99±58 of them were co-registered with scar regions containing iron and 59±28 were from scar regions without iron. The overall mean incidence of ILPs in scar regions containing iron was greater than that from regions without iron (Fig. 9A), but did not reach statistical significance (p = 0.21). Similarly, the mean number of ILPs from regions containing iron, normalized by the percentage of scar volume containing iron, was larger than the mean number of ILPs from regions not containing iron, normalized by percentage of scar volume without iron; and the same observation was made for the mean number of total ILPs normalized by total scar volume (Fig. 9B). However these metrics also did not reach statistical significance (p = 0.12).

## Discussion

In this work, we investigated the effect of chronic iron deposition within infarcted myocardium on the electrical behavior of the heart. From *ex-vivo* electrical measurements in canine myocardium, we showed that one of the consequences of chronic iron deposition within the infarcted zones is the elevation in electrical permittivity (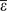) of the infarcted myocardium, which is commensurate with the extent of iron deposition. The bulk electrical measurements also showed that the iron deposition within the infarcted tissue leads to an elevation in electrical permittivity, without a concomitant change in electrical conductivity (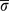). From surface ECG recordings of animals with different *Iron Volume* but similar *Scar Volume*, we found that QT and QTc interval were consistently longer in Iron (>1.5%) group compared to Iron (<1.5%) during the 24-hour period, day, and night; but QTcd was reduced during day, night and the 24-hour period in Iron (>1.5%) group compared to Iron (<1.5%) group. Ultra-high resolution LV endocardial EAMs, co-registered with CMR images, showed a greater incidence of ILPs in regions of scar with iron accumulation than those without iron accumulation. Moreover, there was a tendency for the mean number of ILPs observed per unit volume of scar with iron to be greater than those observed per unit volume of scar without iron or total scar burden.

Biogenic magnetite (hemosiderin) is known to have the highest electrical conductivity of any cellular material [39]. Thus, based on previous reports on composite material properties [19], we expected to find that scar tissue with iron deposits to have a greater 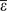 compared to scar tissue without iron (a dielectric). Since the observed changes in 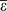 were not accompanied by changes in 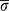, it appears that the effect of iron deposition is to transform the infarcted territory into a capacitor [40]. Currently it is unclear how these alterations in electrical tissue properties mediate the observed changes in surface ECG parameters and EAMs. We anticipate that theoretical modeling may provide mechanistic insight into how or whether these tissue-specific electrical changes mediate alterations in electrical activity in-vivo. Such models need to take into account that the iron deposits following MI are found in the interstitial space and that measurements of 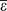 and 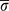 that are reported here were collected in ex-vivo preparations. Moreover, since iron has been known to induce an inflammatory burden on the surrounding myocardium [23], factors other than changes in 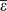 may also explain the electrical changes observed in vivo. Additional studies are needed to explore the mechanism by which iron mediates electrical anomalies in vivo.

We observed the rise in the heart rate during the early morning hours was slower in Iron (>1.5%) dogs than in Iron (<1.5%) dogs (Fig. 7A). In addition, we also found marked prolongation in QT and QTc among Iron (>1.5%) dogs hearts during the day, night and 24 hours compared to Iron (<1.5%) dogs. These differences may be examined in the context of previous studies that attempted to discriminate between sudden cardiac death (SCD) victims and MI survivors in the post infarction period. First, the slow rate of increase in heart rate in SCD victims compared to MI survivors has been reported in the waking hours [41]. Second, marked changes in QTc patterns between SCD victims and MI survivors have also been reported. In fact, prolongation in QTc has been strongly associated with SCD [42]. These collective observations suggest that hearts with significant iron deposition (Iron(>1.5%)) may be more susceptible to arrhythmias than hearts with minimal or no iron (Iron (<1.5%)).

ILPs have been shown to be substrates for ventricular arrhythmias in the post infarction phase [43]–[45]. In particular, ILPs are known to emerge from border zones and within the dense scar [46], [47]. The source of such ILPs has been associated with surviving myocardial bundles within the infarct region, but whether these bundles are indeed the only source of arrhythmias is unclear. In this study, based on CMR and EAM, we demonstrated that dense scar regions with iron deposits have a greater prevalence of ILPs than scarred regions without iron. In addition, this study showed that the number of ILPs per percentage volume of substrate (total scar, scar without iron and scar with iron) is greatest when the substrate is scar with iron. The mechanistic underpinnings contributing to the association between iron accumulations and ILPs in dense scar is not clear. While we speculate that changes in electrical capacitance may explain the isolated potential in the repolarization phase of the cardiac cycle, further studies are necessary to understand how the iron deposits couple with the endogenous electrical activity of the heart to mediate the observed electrical anomalies.

Finally, recent studies [37], [38], [48]–[50] have shown that although infarct volume is a predictor of arrhythmias, the incidence of arrhythmias measured as percentage of arrhythmia-related deaths or implantable cardio-defibrillator (ICD) discharge, plateaus beyond a particular scar volume. Specifically, these studies have shown that the incidence of arrhythmias at a *Scar Volume* of less than 5% to be small, increasing rapidly between 5–10% and then plateauing above 10%. Importantly, these studies showed that the risk of developing arrhythmias increases from 10% (for scar volume <5%) to approximately 40–50% (for scar volume 5–10%). Our analysis showed that for animals within this domain (i.e, 5% < scar volume <10%), it is possible to resolve two groups of dogs with similar scar volumes but with markedly different iron volumes. In fact, our study showed that when animals are dichotomized on the basis of iron volume within this range of scar volumes, the critical ECG parameters of repolarization in animals with markedly higher iron content showed significant prolongation of QT and QTc, as well as shortening of QTcd. Notably, such directional changes, particularly in QTc, have been previously associated with increased risk of ventricular arrhythmias in humans [41], [42]. In addition, it is has been shown that arrhythmogenic substrate is typically found between the subendocardium and the mid-wall [51], [52], an observation that is consistent with the spatial distribution of iron deposits in the chronic infarction. These observations suggest that the extent of iron deposition may be a better predictor of arrhythmias. However, further studies are necessary to translate our findings to the clinical arena.

Collectively taken, our findings from these studies showed that the electrical behavior of infarcts with iron is different from infarcts without iron. These observations point to the plausibility that iron within myocardial scar may serve as a substrate for ventricular arrhythmias in the chronic phase of the infarction period. Additional studies are warranted to understand the mechanism by which iron can potentially mediate arrhythmias. In this respect, it would be of significant interest to understand whether and/or how the iron deposits within the infarcted zones can electrically interact with the surrounding healthy myocardium.

### Limitations

This is the first study to evaluate the effects of iron in the post-infarction scar using ex-vivo measurements, surface ECG recordings and EAM. However, this study has some limitations. First, given the lack of a better method, electrical measurements of the tissue samples isolated from the same slice were weight averaged in order to examine relationships between 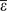 and 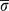 with iron. Implicit in these estimated relationships are that the iron is uniformly deposited within IRON+ infarcts, which may not be accurate. To what extent the heterogeneity of iron deposition affects the local electrical measures remains to be investigated. Topographic current density images of infarcted tissues would be ideally suited to such an investigation. However, the standard conductive atomic force microscopy (C-AFM) typically used for generating current density maps in spatially extended media has practical limitations for use with fragile biological tissues such as the myocardium. Moreover, measurements of 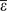 and 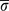 in tissue with and without iron were made from ex-vivo sections, which only account for the extracellular characteristics. Further studies are needed to address the in-vivo effect of iron burden on electrical impedance. Third, this study did not explore mechanistic insight into how the iron deposition in the post infarction period mediates electrical anomalies. Additional studies are needed to explore this limitation. Fourth, the surface ECG recordings were limited to a 24-hour period but longer recordings have the potential to further confirm our 24-hour observations. In addition, while the metrics used to evaluate the mean difference in the overall incidence of ILPs and ILP count relative to substrate burden tended to be greatly influenced by the presence and extent of iron deposits, they did not reach statistical significance, likely because of small sample size. Studies with larger sample size may establish statistical significance. Finally, while this study demonstrated the possibility that iron depositions in the chronic infarction induce electrical anomalies in dogs, its effect on the electrical behavior in humans following myocardial infarction remains to be investigated. However, if these findings can be extended to humans, iron may evolve as a novel arrhythmogenic substrate and T2* CMR can be used to noninvasively guide appropriate patient selection for ICD placements.

## Supporting Information

File S1 **Estimation of tissue conductivity and permittivity.**
(DOCX)
