# Supplementary material for: Iron Deposition following Chronic Myocardial Infarction as a Substrate for Cardiac Electrical Anomalies: Initial Findings in a Canine Model
Source: PLoS One. 2013 Sep 16;8(9):e73193. doi: 10.1371/journal.pone.0073193 (PMC3774668; doi:10.1371/journal.pone.0073193)
Supplement: File S1 — Estimation of tissue conductivity and permittivity. (DOCX) [file pone.0073193.s001.docx]

**S1 Supporting Material**

***Estimation of tissue conductivity and permittivity***

Bulk electrical permittivity and conductivity of each sample were derived from the AC-impedance measurements. Surface area (*A* in m^2^) and distance between the electrodes (*d* in m) after the sample is placed between the electrodes were measured. The complex admittance *Y* (in siemens S) of the sample was calculated as the reciprocal of *Z*, which can be further expressed as follows^29^

$$Y=G+i\omega C$$

where *G* is the conductance (in S), *C* is the capacitance (in F), *ω* is the angular frequency (in rad/s) and *i* is $\sqrt{-1}$. *G* and *C* can be further expressed as follows

$$G=\frac{A\times\sigma}{d}$$

$$C=\frac{A\times\varepsilon}{d}$$

where $\sigma$and $\varepsilon$are bulk conductivity (in S/m) and permittivity (in F/m) respectively. Bulk $\sigma$and$\varepsilon$ of each sample were therefore calculated from the original complex impedance data (*Z*) as follows

$$\sigma=\frac{\mathrm{Re}\left(\frac{1}{Z}\right)\times d}{A}$$

$\varepsilon=\frac{\mathrm{Im}\left(\frac{1}{Z}\right)\times d}{A\times\omega}$.
